# Predictive and Prognostic Biomarkers for Lung Cancer Bone Metastasis and Their Therapeutic Value

**DOI:** 10.3389/fonc.2021.692788

**Published:** 2021-10-14

**Authors:** Xupeng Chai, Eloy Yinwang, Zenan Wang, Zhan Wang, Yucheng Xue, Binghao Li, Hao Zhou, Wenkan Zhang, Shengdong Wang, Yongxing Zhang, Hengyuan Li, Haochen Mou, Lingling Sun, Hao Qu, Fangqian Wang, Zengjie Zhang, Tao Chen, Zhaoming Ye

**Affiliations:** ^1^ Department of Orthopedics, Musculoskeletal Tumor Center, The Second Affiliated Hospital of Zhejiang University School of Medicine, Hangzhou, China; ^2^ Institute of Orthopedic Research, Zhejiang University, Hangzhou, China

**Keywords:** predictive/prognostic biomarkers, lung cancer, bone metastasis, bone formation-associated markers, bone resorption-associated markers, bone metastasis-associated signaling markers

## Abstract

Lung cancer is the leading cause of cancer-related death worldwide. Bone metastasis, which usually accompanies severe skeletal-related events, is the most common site for tumor distant dissemination and detected in more than one-third of patients with advanced lung cancer. Biopsy and imaging play critical roles in the diagnosis of bone metastasis; however, these approaches are characterized by evident limitations. Recently, studies regarding potential biomarkers in the serum, urine, and tumor tissue, were performed to predict the bone metastases and prognosis in patients with lung cancer. In this review, we summarize the findings of recent clinical research studies on biomarkers detected in samples obtained from patients with lung cancer bone metastasis. These markers include the following: (1) bone resorption-associated markers, such as N-terminal telopeptide (NTx)/C-terminal telopeptide (CTx), C-terminal telopeptide of type I collagen (CTx-I), tartrate-resistant acid phosphatase isoform 5b (TRACP-5b), pyridinoline (PYD), and parathyroid hormone related peptide (PTHrP); (2) bone formation-associated markers, including total serum alkaline phosphatase (ALP)/bone specific alkaline phosphatase(BAP), osteopontin (OP), osteocalcin (OS), amino-terminal extension propeptide of type I procollagen/carboxy-terminal extension propeptide of type I procollagen (PICP/PINP); (3) signaling markers, including epidermal growth factor receptor/Kirsten rat sarcoma/anaplastic lymphoma kinase (EGFR/KRAS/ALK), receptor activator of nuclear factor κB ligand/receptor activator of nuclear factor κB/osteoprotegerin (RANKL/RANK/OPG), C-X-C motif chemokine ligand 12/C-X-C motif chemokine receptor 4 (CXCL12/CXCR4), complement component 5a receptor (C5AR); and (4) other potential markers, such as calcium sensing receptor (CASR), bone sialoprotein (BSP), bone morphogenetic protein 2 (BMP2), cytokeratin 19 fragment/carcinoembryonic antigen (CYFRA/CEA), tissue factor, cell-free DNA, long non-coding RNA, and microRNA. The prognostic value of these markers is also investigated. Furthermore, we listed some clinical trials targeting hotspot biomarkers in advanced lung cancer referring for their therapeutic effects.

## Introduction

Lung cancer, comprising non-small-cell lung cancer (NSCLC) and small-cell lung cancer (SCLC), is the leading cause of cancer-related death worldwide, with approximately 1.6 million deaths reported annually (1). Approximately 36% of all lung cancers and 54.5% of the stage II–IIIA NSCLC cases showed postoperative recurrence or metastasis. Bone metastasis, which is the most common site for tumor distant dissemination, is detected in more than one-third of patients with advanced lung cancer (2, 3). Patients with bone metastases experience complications, including severe bone pain, pathological fractures, spinal cord and nerve compression, and other skeletal-related events, which significantly reduce patient quality of life (2, 4). According to the guidelines of the European Society for Medical Oncology and several clinical studies, use of bisphosphonates (e.g., zoledronic acid) or anti-receptor activator of nuclear factor κB (NF-κB) ligand (anti-RANKL) monoclonal antibody (e.g., denosumab) is recommended for the treatment of lung cancer patients with bone metastasis (5–9).

The detection of lesions in patients at early disease stages before the occurrence of distinct clinical symptoms is challenging. This delayed diagnosis shortens the optimal treatment period. Therefore, early diagnosis of bone metastasis at the initial staging work-up could lead to markedly improved clinical outcomes. Thus far, the most accurate method for the detection of bone metastasis is bone marrow biopsy. Moreover, imaging diagnostic methods, including X-ray, computed tomography, magnetic resonance imaging, technetium 99m-methyl diphosphonate (^99m^Tc-MDP) bone scan, and ^18^F-fluorodeoxyglucose positron emission tomography/computed tomography play critical roles (10, 11). However, the clinical application of these procedures is limited due to their low specificity, invasive operation, complex process, and high costs. Thus, other indirect markers are unavoidably required for predicting lung cancer bone metastasis in addition to its prognosis.

It is widely acknowledged that adenocarcinoma, pathological stage III disease, and advanced age are significantly related to a high risk of bone metastasis (12–14). Moreover, lymph node metastasis has been associated with higher odds of multiorgan metastases and a worse overall survival (OS) (15). Programmed cell death 1 ligand 1 (PD-L1) immunochemistry is commonly used as a prognostic biomarker of immune checkpoint inhibitor for clinical decision making. Nevertheless, some researchers suggest that the usefulness of PD-L1 expression is controversial (16–18). Moreover, the tumor mutational burden is measured by whole exome and next-generation sequencing to predict the clinical outcome after treatment with immune checkpoint inhibitor, as well as bone metastasis (19). Nevertheless, these factors provide limited information. Thus, it is important to investigate novel methodologies and therapeutics that would increase the proportion of patients responding to such treatments, while simultaneously improving their side effect profile.

Studies on potential markers in the serum, urine, and tumor tissue were undertaken to predict the bone metastases in patients with NSCLC. Some of these markers are stable and easy to detect, and potentially helpful in disease classification, diagnosis, and treatment.

**Figure 1:**
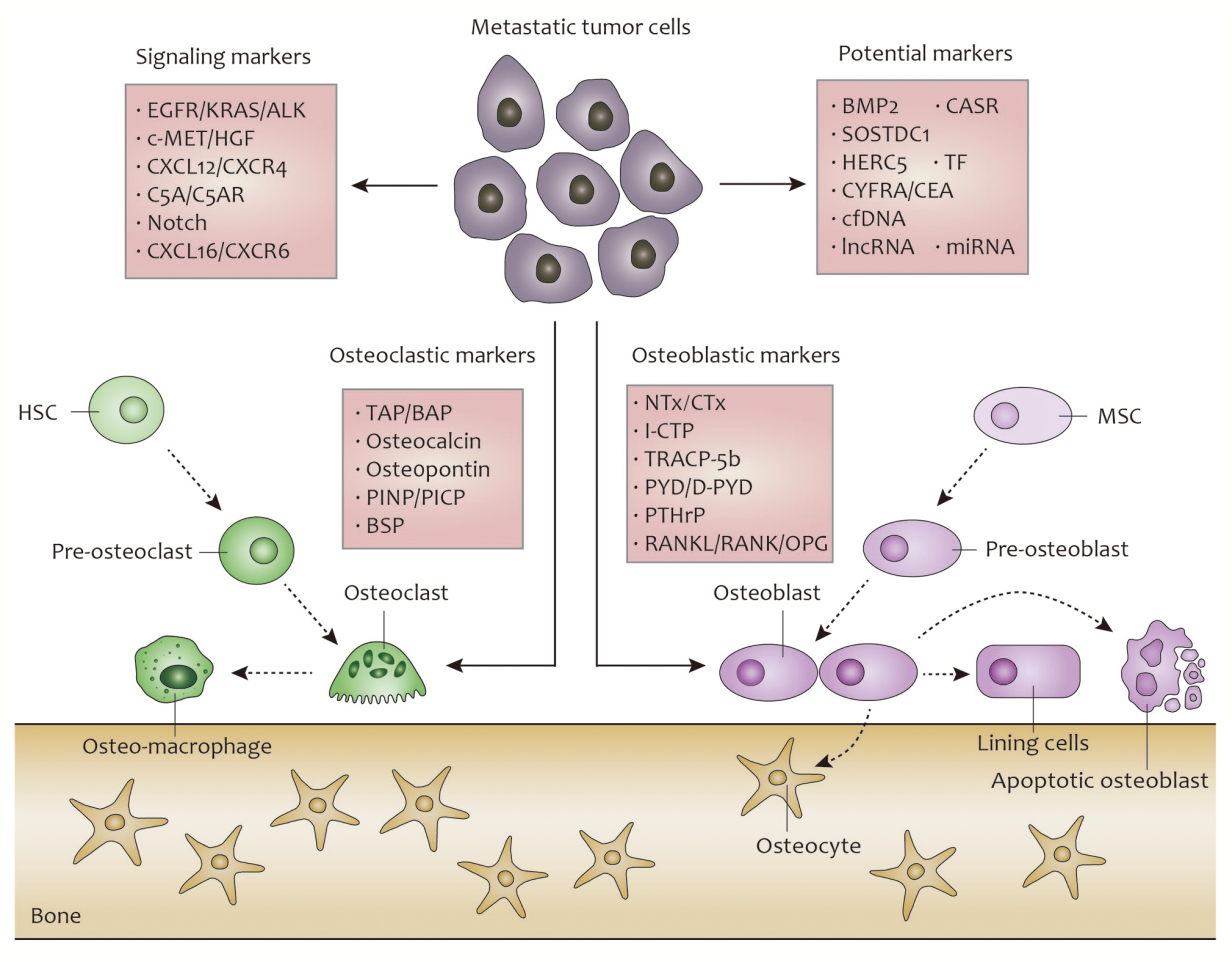
Overall concept picture about biomarkers during the progress of lung cancer bone metastasis.

In this review, we summarize the findings of recent clinical research studies on markers detected in samples obtained from patients with lung cancer, and analyze their capacity for the prediction of treatment response and prognosis of bone metastasis. For a logical classification, these markers are divided into bone resorption-associated markers, bone formation-associated markers, bone metastasis-associated signaling markers, and others (20). The overall concept pictureof lung cancer bone metastasis is showed in **Figure 1**.

## Osteocalst- and Bone Resorption-Associated Biomarkers

The components of the bone microenvironment, including osteocytes, immune cells, and bone matrix, play critical roles in bone metabolism and tumor cell seeding, dormancy, and growth (21). The interaction between osteoclasts and osteoblasts promotes bone degradation through the release of several factors. Disseminated lung cancer cells activate osteoclasts around metastatic foci, which facilitate expansion in the mineralized matrix (22). During these processes, the rate of resorption can also be quantified through several bone resorption markers, which are described below.

### N-terminal Telopeptide (NTx) and C-Terminal Telopeptide (CTx)

Type I collagen is the principal collagen in the skeletal system, accounting for approximately 90% of the organic chemical constituents of bone. NTx and CTx are the degradation products of collagen which are released into blood when bone is absorbed, and subsequently collected in urine after kidney drainage (23, 24). Studies performed by Izumi et al. and Chung et al. indicated that urinary NTx plays a significant role in the diagnosis of patients with bone-metastatic lung cancer (25, 26). In addition, the levels of serum NTx were increased in patients with NSCLC as well as other solid tumors with bone metastasis (23, 27). Bayrak et al. (28) demonstrated that, at the threshold value of 25.69 nmol bone collagen equivalents (BCE), the sensitivity of serum NTx was 90.42% and the specificity was 43.4%. Kaira et al. (29) revealed that normalization of elevated urinary NTx at 1 month after chemotherapy was correlated with the therapeutic response of patients with bone-metastatic lung cancer. Moreover, Zhang et al. (30) analyzed 1,279 patients and revealed that serum concentration of NTx was significantly high in the Chinese population with bone metastasis from solid tumors including lung cancer. Similarly, the measurement of CTx is considered very helpful for the complementary diagnosis of bone metastasis from lung tumor (31–33). However, some researches indicated that the levels of CTx were not significantly different between patients and healthy individuals (28). These inconsistent results may be attributed to the effects of diets or injuries on the levels of CTx. Therefore, both NTx and CTx are beneficial in detecting bone metastasis in lung cancer and worth further exploring.

### C-Terminal Telopeptide of Type I Collagen (I-CTP)

Pyridinoline (PYD) cross-linked I-CTP is a metabolite released during the degradation of type I collagen. It is recognized as one of the most accurate markers for bone metastasis. In 2005, Yokoyama et al. (34) conducted a clinical research study of 87 patients with primary lung cancer, showing that serum I-CTP was significantly higher in patients with bone metastasis than those without. The most efficient cut-off value for I-CTP computed in this study was 6.4 ng/ml. A study performed by Tanaka et al. (35) in 2013, which included a larger number of patients, yielded similar results. Charpko et al. (36) utilized the ^99m^Tc-MDP scan technique to directly demonstrate the correlation between serum I-CTP and the extent of metastatic disease in bone. Kong et al. (37) compared the clinical effect of serum β-type collagen carboxy-terminal peptide and I-CTP in 126 patients, demonstrating that the latter has higher sensitivity and accuracy with a similiar receiver operating characteristic curve [area under the curve (38)] value. Tang et al. (33) combined tartrate-resistant acid phosphatase isoform-5b (TRACP-5b) and I-CTP as prognostic factors, revealing an elevated AUC of 0.895 compared with that of each factor alone (p<0.0001); the cut-off values were 7.6 U/l and 8.4 U/l, respectively. These studies showed that I-CTP may be a reliable marker and assessment using several markers can be more beneficial in the prediction of bone metastasis.

### TRACP-5b

TRACP-5b is an enzyme secreted mainly by activated osteoclasts, thus reflecting the enrichment and vitality of osteoclasts. Its serum levels reflect the degree of osteolytic bone metastasis and the tumor burden within the bone milieu (39). Moreover, this enzyme responds rapidly and significantly to anti-resorptive treatment and is minimally affected by diet and renal or hepatic disease compared with NTx and CTx. These features render TRACP-5b to be a bone-specific assessment factor. Studies have demonstrated that the levels of TRACP-5b are significantly higher in breast cancer and prostate cancer with bone metastasis (40, 41). Notably, in patients with lung cancer bone metastasis, some clinical research has produced controversial results regarding its accuracy (32, 33, 42–44). In clinical cases, the elevation of TRACP-5b can be caused by physiological processes, such as osteoporosis and hyperparathyroidism. Thus, other markers should be considered in combination with TRACP-5b for prediction.

### Pyridinoline(PYD) Cross-Linked and Deoxypyridinoline (D-PYD) Cross-Linked

Serum PYD cross-linked is a cross-linked amino acid appearing in the collagen composition of numerous tissues, which could be deoxidized into D-PYD cross-linked. PYD and D-PYD are released into the circulation as a result of osteoclastic degradation of the bone matrix (45, 46). Ebert et al. (42) stated that the levels of serum PYD and D-PYD were higher (p<0.01) in patients with bone metastasis than those with benign lung diseases. The sensitivity (specificity) values were 91.8% (24.1%) and 83.7% (34.5%), respectively. Furthermore, Dane et al. (47) revealed that the high levels of urinary D-PYD may be an early sign of metastasis in patients without bone abnormality assessed by imaging techniques. This is because D-PYD is not internally metabolized, but excreted in the urine. Hence, the measurement of D-PYD in urine is of great importance in monitoring bone resorption.

### Parathyroid Hormone-Related Peptide (PTHrP)

PTHrP, secreted by malignant tumor cells, acts as a potent stimulator of osteoclastic bone resorption and may induce hypercalcemia (48). Miki et al. (49) designed a series of studies with anti-PTHrP neutralizing antibody. They found that PTHrP played an essential role in bone metastasis, but not visceral metastases, in SCLC, indicating to some extent the specificity of PTHrP. Katseli et al. (50) collected the circulating tumor cells in peripheral blood and concluded that the mRNA levels of PTHrP correlated positively with lung cancer stage, presence of bone metastasis, and squamous cell carcinoma. Deng et al. (51) detected the upregulated expression of PTHrP in bone metastasis sites of NSCLC by reverse transcription-polymerase chain reaction(RT-PCR) and immunohistochemistry. Teng et al. (52) constructed a novel model, including PTHrP, for the early identification of lung cancer bone metastasis. This model included 205 patients, and the average predictive time for the occurrence of bone metastasis was 9.46 months (shorter than that of bone scan diagnosis). Moreover, the response to some osteoclast-targeted therapies, including erlotinib and reveromycin A, could also be estimated by the serum levels of PTHrP (53, 54).

### RANKL/RANK/Osteoprotegerin (RANKL/RANK/OPG) System

The RANKL/RANK/OPG pathway is a vital regulator of bone metabolism in normal and pathological conditions, such as the “Vicious Cycle” (55). RANK is a surface receptor specifically expressed on osteoclasts and their progenitors. It is activated by binding to RANKL expressed on the surface of osteoblasts and stromal cells, with the assistance of macrophage-colony stimulating factor (M-CSF) (56). The RANK/RANKL interaction subsequently activates transcription factors, such as NF-κB, and eventually increases the number of activated osteoclasts (57). OPG, a decoy receptor secreted by osteoblasts and stromal cells, inhibits the positive effects of RANKL on osteoclasts by preventing the RANKL/RANK interaction. This leads to hindrance of osteoclastogenesis and bone resorption (58).

The RANKL/RANK/OPG axis has been used in the prediction of bone metastasis. Karapanagiotou et al. (43) demonstrated that increased serum levels of RANKL were detected in NSCLC patients with bone metastases, while increased levels of OPG were observed in all patients with lung cancer. Terpos et al. (44) revealed an unusually low serum TRACP-5b/RANKL ratio in patients with existing or subsequent metastasis. Santini et al. (59) performed an immunohistochemical analysis of tumor tissues collected from 74 patients. The results displayed that 89% of the patients with bone metastasis were RANK-positive, and 59.5% of patients showed >50% positive tumor cells. Moreover, there was no significant difference noted in the expression of RANK between primary tumors and metastases (59). According to these research studies, the RANK/RANKL pathway shows great stability and reliability in the diagnosis of bone metastases of lung cancer, and RANKL-targeting agents could serve as a rational approach for the prevention of bone metastases (60, 61).

The therapeutic effect of RANK-targeting denosumab in the treatment of advanced breast cancer, prostate cancer, and multiple myeloma was demonstrated in a series of phase III studies (62–65). Recently, a randomized, open-label, phase III SPLENDOUR trial was designed to evaluate the treatment of denosumab in patients with advanced lung cancer. The addition of denosumab to chemotherapy in the intent-to-treat population and subgroups with or without bone metastases did not lead to improvement in OS (66). Of note, denosumab was well-tolerated, without unexpected safety concerns. Clinical trials of denosumab that are currently recruiting patients are listed in **Table 5**. Moreover, some studies revealed the effect of serum RNA levels of RANK, RANKL, and OPG in the evaluation of zoledronate acid therapy in patients (61). Additionally, the use of thrombospontin was considered for the prevention of osteoporosis and bone metastases (67).

## Osteoblast- and Bone Formation-Associated Biomarkers

Lung cancer cells implanted in bone loci also affect the activity of osteoblasts in the microenvironment. Osteoblasts are activated by a large amount of growth factors and mediated by certain osteoclast-secreted proteins, which could to some extent predict the skeletal invasion of cancer cells (22).

### Total Serum Alkaline Phosphatase/Bone Specific Alkaline Phosphatase (TAP/BAP)

TAP is widely distributed in the body with a series of isoenzymes. TAP lacks diagnostic specificity because of its sensitivity to several non-neoplastic diseases, such as liver cancer or cholecystitis. 68] Thus, few research studies focus on TAP. Instead, BAP is thought to be a reliable marker reflecting osteoblast activity and mineralization as one of the isoenzymes of TAP. Aruga et al. (68) and Ebert et al. (42) proved that the concentrations of TAP and BAP were significantly higher in patients with bone metastasis than those without (p<0.01). Lumachi et al. (69) proposed that there is no correlation between age and BAP (R=−0.10, p=0.61) among patients with bone metastases. Bayrak et al. (28) obtained a BAP threshold value of 22.38 μg/l with sensitivity of 60.87% and specificity of 69.05%. Similarly, Tang et al. (33) obtained cut-off values of 21.8 μg/l with an AUC of 0.760. Notably, there are two age-dependent physiological peaks of serum BAP at infancy and puberty. These peaks in levels are associated with altered longitudinal growth and may affect the reliability of results (70). These studies revealed that BAP plays an important role in predicting bone metastasis of lung cancer, while its clinical use require more limitation, such as age.

### Osteocalcin (OC)

OC is the most abundant non-collagenic protein in the bone extracellular matrix. It is only produced by osteoblasts and odontoblasts under the control of vitamin D3 (71, 72). OC and fragments of the peptide are released during bone resorption, indicating its potential role as a marker of bone metastasis. Karapanagiotou et al. (43) and Terpos et al. (44) demonstrated that decreased OC serum levels were detected in NSCLC patients with bone metastases. In contrast, Bayrak et al. (28) argued that the levels of OC were not significantly different between the bone metastasis and no metastasis groups. Therefore, considering its limited accuracy and stability, OC may not be an ideal choice for predicting bone metastasis.

### Osteopontin (OPN)

OPN is a sibling glycoprotein first identified in osteoblasts. It is defined as an extracellular matrix protein, which plays a vital role in the mineralization and absorption of bone matrix (72). In the other side, OPN regulates the migration and adhesion of tumor cells by binding to its receptor CD44. A study confirmed that OPN is overexpressed in NSCLC tumor tissues compared with their adjacent normal counterparts, and significantly correlated with TNM stages and lymph metastasis (73). Karapanagiotou et al. (43) and Terpos et al. (44) found increased serum levels of OPN in NSCLC patients with bone metastases. Moreover, Zhen et al. (74) performed a study of 105 patients and constructed a molecular model utilizing four serum biomarkers. As a potential therapeutic target, OPN with different genotypes was further investigated. Chen et al. (75) also compared the bone metastasis rate of three promoter OPN polymorphisms, namely the −OPN-66T/G, −156G/GG, and −443C/T variants, from the DNA of blood lymphocytes. They declared that patients with the −443 (CC) variant had a significantly higher incidence of bone metastasis compared with the other genotypes.

CD44 is a cell-surface receptor for OPN and mediates epithelial-mesenchymal transition; hence, it is related to tumor migration (76). Liu et al. (77) genotyped the CD44 gene in DNA extracted from blood lymphocytes of patients with NSCLC and found that the rs187115 (AG+GG) genotype was a significant predictor of bone metastasis and poor survival. It is widely accepted that the detection of single nucleotide polymorphisms in CD44 and other genes would be helpful in assessing the invasion ability of tumors (78).

### Amino-Terminal Extension Propeptide of Type I Procollagen/Carboxy-Terminal Extension Propeptide of Type I Procollagen (PINP/PICP)

PICP and PINP are produced by the extracellular cleavage of a molecule of type I procollagen at the moment of its incorporation into the bone matrix (71). The concentrations of PICP and PINP, reflecting the rate of synthesis of type I collagen, are altered by the liver where they are degraded. Kobayashi et al. (79) demonstrated that the serum levels of PINP were significantly correlated with the clinical stage, extent of bone metastasis, survival time, levels of D-dimer, and tumor size in lung cancer. In 2004, Elbert et al. (42) demonstrated that the concentration of PINP was significantly higher in patients with bone metastasis than those without (p<0.01), with sensitivity and specificity of 18.4% and 97.5%, respectively. Other researchers, including Lüftner et al. (80), assessed the predictive effect of the serum levels of PINP on the osseous spread of breast and prostate cancer, while studies on lung cancer are limited and require more attention.

### Bone Sialoprotein (BSP)

BSP, a sulfated phosphoprotein enriched in bone and other mineralized tissue, is critical in elucidating the formation of bone metastases (81). In 1997, Bellahcene et al. (82) found that BSP was not detected in normal lung tissue, except the cartilage in bronchi. On the contrary, most of the adenocarcinomas and all squamous carcinomas among the 86 tested lung tissues had detectable levels of BSP. Moreover, Zhang et al. (83) further declaimed that BSP expression in patients with tumor-resected NSCLC is significantly correlated with bone metastasis and can be utilized to identify high-risk patients after primary tumor resection. Similarly, He et al. (84) revealed that the serum levels of BSP could be used to evaluate bone metastasis, with a cut-off value of 33.56 ng/ml, sensitivity of 77.8, and specificity of 81.1%. Collectively, these findings indicated that BSP is a stable component of a bone metastasis predictive model, like in the study conducted by Zhou et al. (74).

## Bone Metastasis-Associated Signaling Markers

Osteoclast and osteoblast functions are thought to be essential in bone metastasis for different types of lung cancer, and varying degrees of osteolytic and osteoblastic activities have been observed. As expected, more potential markers are involved in the progression of osteolytic and osteoblastic metastasis, by mediating downstream proteins or the signaling axis. These markers should be taken into consideration for the early diagnosis of lung cancer bone metastasis, although the mechanisms involved in this process remain unknown (81).

### Epidermal Growth Factor Receptor/Kirsten Rat Sarcoma/Anaplastic Lymphoma Kinase (EGFR/KRAS/ALK) Axis

Lung cancer is often driven by molecular alterations, such as EGFR and KRAS mutations, and ALK rearrangements expressed in tumor tissues of patients with NSCLC. Fujimoto et al. (85) demonstrated that patients with EGFR mutations (N=98) had significantly more metastatic lesions in the brain and bone than those in the wild-type group. In addition, EGFR mutations were significantly more frequent in patients with multiple lung metastases than a single lung metastasis (24/40 *vs.* 12/42, respectively; p=0.004). Kuijper et al. (86) found that EGFR+ tumors were linked to more frequent metastases to the bone (31.5% *vs.* 53.8%, respectively) and pleura (24.1% *vs.* 37.5%, respectively) compared with triple-negative tumors. Of note, Hendriks et al. (87) and Dormieux et al. (88) showed that the incidences of brain and bone metastases were not different between EGFR+, KRAS+, and wild-type patients. Nevertheless, Zang et al. (13) and Amelot et al. (89) revealed that OS was associated with the EGFR status in patients with NSCLC spinal metastasis.

EGFR mutations may also be a useful prognostic factor for the response to EGFR tyrosine kinase inhibitors (TKIs) (90). The majority of these mutations are located in the catalytic kinase domain of EGFR. Typical mutations include a deletion within exon 19 (E19del) or a leucine-to-arginine point mutation at codon 858 (L858R) within exon 21 (91). The detection of E19del and L858R by immunohistochemistry predicts the effectiveness of first-generation EGFR TKIs (e.g., gefitinib and erlotinib) and second-generation TKIs (e.g., afatinib) (92, 93). Other studies identified T790M mutations in EGFR exon 20 as predictors for the development of resistance to first-generation EGFR TKIs (94–96). According to the phase III AURA-3 trial, treatment of patients with T790M mutations using osimertinib resulted in a better clinical outcome compared with chemotherapy, with an objective response rate of 71% *versus* 31%, respectively, and median progression-free survival (PFS) of 10.1 *versus* 4.4 months, respectively (97). In the phase III FLAURA study involving 556 patients with untreated EGFR-mutated NSCLC, osimertinib significantly enhanced the objective response rate, median PFS, and median OS compared with gefitinib or erlotinib (98, 99). Importantly, 49% of patients lost the T790M mutation during treatment with osimertinib, which predicted their resistance to osimertinib and worse outcome. This mutation is usually associated with the emergence of competing resistance mechanisms, such as KRAS mutations, c-MET amplification, small-cell transformation, and gene fusion (100, 101). Furthermore, mutations (e.g., L792, L718, G719, and exon 20 insertions) may predict exceedingly poor responses to osimertinib and standard TKIs; however, their exact prognostic and predictive roles are not fully clarified (102–104). Some potential TKIs targeting these mutations, including roziotinib and TAK-788, remain under clinical investigation (**Table 1**). It is assumed that the EGFR mutations of primary lung cancer could forecast the prognosis of patients with bone metastasis and tumor response to TKI therapy.

**Table 1:** Studies on bone resorption markers.

Marker	Reference	Sample location	Sample size (N)	Finding
**NTx and CTx**	Jablonka et al. (105)	Serum	181	Serum NTx levels were significantly higher in patients with solid tumor and bone metastasis (BM) than in those without BM and in normal controls.
	Kaira et al. (106)	Urine	30	In 30 patients, the median NTx levels at 1 month after one cycle of chemotherapy were significantly lower than those at baseline (p=0.0016).
	Bayrak et al. (107)	Serum	65	At the threshold value of 25.69 nmol BCE, the sensitivity of BM diagnosis of NTx was 90.24% and the specificity was 43.4%.
	Huang et al. (108)	Serum	1,720	NTx was significantly lower among patients with BM than those without BM.
	Zhang et al. (106)	Serum	1,279	There was a significant relationship between serum NTx concentration and BM from solid tumors in the Chinese population.
**I-CTP**	Yokoyama et al. (34)	Serum	87	I-CTP was significantly higher in patients with BM than those without BM. The most efficient cut-off value for I-CTP computed in this study was 6.4 ng/ml.
	Charpko et al. (36)	Serum	60	The levels of bone pathological degradation (I-CTP) and bone formation reflected the extent of metastatic disease in bone measured by ^99m^Tc-MDP scans.
	Kong et al. (37)	Serum	126	The levels of both β-CTX and I-CTP were significantly higher in lung cancer patients with BM (both p<0.001) and ICTP had a better sensitivity and accuracy than β-CTX.
	Tanaka et al. (35)	Serum	143	The rate of distant metastasis was significantly higher in patients with elevated preoperative serum I-CTP levels than those with normal preoperative I-CTP levels (≤4.5 ng/ml) (p<0.0001)
**TRACP-5b**	Ebert et al. (41)	Serum/urine	146	There was no significant difference in TRACP-5b activity between lung cancer patients with BM *versus* those without. Power as a diagnostic marker is low. Does not correlate with the extent of BM.
	Terpos et al. (43)	Serum	79	Elevated TRACP-5b at diagnosis increased the risk of disease progression.
	Karapanagiotou et al. (42)	Serum	68	There was no significant difference between patients with lung cancer with or without BM and the control group.
	Tang et al. (33)	Serum	265	The TRACP-5b value was higher in patients with BM than those without BM (p<0.0001).
**PYD, D-PYD**	Ebert et al. (41)	Serum	150	PYD and D-PYD were higher (p<0.01–0.05) in patients with BM. The sensitivity (specificity) values were 91.8% (24.1%) and 83.7% (34.5%) for PYD and D-PYD, respectively.
	Dane et al. (46)	Urine	60	The high urinary levels of D-PYD may be an early sign of metastases in patients without BM assessed by scintigraphic techniques.
**PTHrP**	Katseli et al. (49)	Circulating tumor cells	125	PTHrP mRNA detection correlated positively with lung cancer stage, presence of BM, and squamous cell carcinoma.

AUC, area under the curve; BCE, bone collagen equivalents; β-CTX, beta-type collagen carboxy-terminal peptide; CTx, C-terminal telopeptide; D-PYD, deoxypyridinoline; I-CTP, C-terminal telopeptide of type I collagen; NTx, N-terminal telopeptide; TRACP-5b, tartrate-resistant acid phosphatase isoform-5b; PTHrP, parathyroid hormone related peptide; PYD, pyridinoline.

The ALK rearrangement is present in approximately 8% of NSCLC patients with oncogenic drivers (109). Dormieux et al. (88) demonstrated that the group with ALK fusions developed more brain and lung metastases compared with the negative group, with rates of 42% *versus* 29% and 37% *versus* 24%, respectively. Despite the absence of a direct correlation between ALK fusions and bone metastasis, the detection of these rearrangements contributes to the classification of target therapies for lung cancer patients with bone metastasis. The phase III PROFILE 1014 trial compared crizotinib with chemotherapy and demonstrated better objective response rate and PFS in ALK-positive patients (110). Moreover, the second-generation ALK-TKIs ceritinib, alectinib, and brigatinib, and the third-generation ALK TKI lorlatinib, showed an enhanced effect on the treatment of ALK-rearranged NSCLC compared with crizotinib. A variety of secondary ALK point mutations (e.g., L1196M, G1202R, S1206Y, and 1151Tins) were detected in patients with progressed disease, some of which predicted resistance to crizotinib (111–113). Recent clinical trials are presented in **Table 2**.

**Table 2:** Studies on bone formation markers.

Marker	Reference	Sample location	Sample size (N)	Finding
**BAP/TAP**	Lumachi et al. (57)	Serum	35	Using a cut-off value of 50 U/l for BAP, the sensitivity and specificity were 37.5% and 84.2%, respectively.
	Bayrak et al. (28)	Serum	65	Serum levels of total ALP and BAP were significantly higher in the group with bone metastasis (p<0.05). According to the ROC curve analysis, at the threshold value of 22.38 μg/l, the sensitivity and specificity of BAP were 60.87% and 69.05%, respectively.
	Tang et al. (33)	Serum	143	The area under the ROC curve (AUC) of BAP was 0.760 (p<0.0001). The cut-off value for BAP was 21.8 μg/l.
**Osteocalcin (OC)**	Karapanagiotou et al. (42)	Serum	68	Decreased OC and increased OPN serum levels were detected in NSCLC patients with bone metastasis.
	Terpos et al. (43)	Serum	79	OC was significantly decreased in the group of patients who developed bone metastasis at some point during the course of their disease. Patients with bone metastasis showed an increase in OPN.
	Bayrak et al. (28)	Serum	65	OC was not significantly different between the group with and without bone metastasis.
**Osteopontin (OPN)**	Chen et al. (63)	Blood lymphocytes	360	Patients with the OPN−443 (CC) variant had a significantly higher incidence of bone metastasis compared with other genotypes.
**PINP/PICP**	Kobayashi et al. (67)	Serum	59	PINP was significantly correlated with clinical stage, extent of bone metastasis, survival time, D-dimer, and tumor size.
	Ebert et al. (41)	Serum	150	The concentration of PINP was significantly higher in patients with bone metastasis (p<0.01). The sensitivity (specificity) values for PINP and PICP were 18.4% (97.5%) and 2.1% (95.2%), respectively.

ALP, alkaline phosphatase; BAP, bone specific alkaline phosphatase; NSCLC, non-small-cell lung cancer; PICP, carboxy-terminal extension propeptide of type I procollagen; PINP, amino-terminal extension propeptide of type I procollagen; ROC, receiver operating characteristic; TAP, total serum alkaline phosphatase.

KRAS mutations are among the most commonly found mutations in NSCLC (20–30% of cases) (114–116). Its mutations mainly occur in codons 12, 13, and 61, which are thought to be diagnostic factors of bone metastasis of lung cancer. Renaud et al. (117) claimed that patients with KRAS G12C rearrangement developed significantly more bone metastases compared with those in the control group of the cohort (59% *vs.* 16%, respectively; p<0.0001) after thoracic surgery for NSCLC. Currently, there are two investigational KRAS G12C inhibitors, namely AMG 510 and MRTX849 (**Table 3**), which have shown 40–50% response rate in some pre-treated patients (106, 123, 124). Further investigation of other specific therapies may reveal that KRAS mutations could also be useful for the classification of KRAS-targeted therapy.

**Table 3:** Current studies on signaling markers.

Marker	Reference	Sample location	Sample size (N)	Finding
**EGFR/KRAS/ALK**	Fujimoto et al. (72)	Tumor tissue	276	The EGFR-mutated group had significantly more metastatic lesions in the brain and bone than the wild-type group. EGFR mutations were significantly more frequent in patients with multiple lung metastases than those with a single lung metastasis.
	Hendriks et al. (74)	Tumor tissue	189	The incidence of brain and bone metastases was not different between EGFR+, KRAS+, and wild-type patients. Post-metastatic bone disease survival was significantly longer in EGFR+ patients.
	Renaud et al. (100)	Tumor tissue	481	After thoracic surgery for NSCLC, patients with KRAS G12C developed significantly more bone metastases compared with the remainder of the cohort (59% *vs.* 16%, respectively; p<0.0001). More patients with mEGFR developed liver and brain metastases (30% *vs.* 10%, respectively; p=0.006; 59% *vs.* 1%, p<0.0001, respectively). Patients with KRAS G12V developed significantly more pleuro-pericardial metastases (94% *vs.* 12%, respectively; p<0.0001).
	Kuijpers et al. (73)	Tumor tissue	1,994	Compared with triple-negative tumors, EGFR+ tumors had more metastases to the bone (31.5% *vs.* 53.8%, respectively) and pleura (24.1% *vs.* 37.5%, respectively). At diagnosis, KRAS+ and ALK+ tumors had metastasized more frequently to the lungs (20.3% *vs.* 26.7%, respectively) and liver (13.1% *vs.* 23.8%, respectively).
	Zang et al. (118)	Tumor tissue	176	In patients with NSCLC spinal metastasis, survival was associated with the EGFR status and many other factors.
	Amelot et al. (76)	Tumor tissue	210	In patients with NSCLC spinal metastasis, ALK gene rearrangement (p<0.0001) and mEGFR (p<0.0001) were associated with longer survival.
	Dormieux et al. (75)	Tumor tissue	550	EGFR-mutated tumors preferentially spread to the pleura and less commonly to the adrenals. ALK-rearrangement tumors usually spread to the brain and the lungs, whereas BRAF-mutated tumors are unlikely to spread to bones (21% *vs.* 42%, respectively; p=0.011).
	Yao et al. (104)	Tumor tissue	75	With F-FDG PET/CT, low bmSUVmax is more frequently observed in EGFR mutations, rendering it an independent predictor of these mutations.
	Terpos et al. (43)	Serum	79	Patients with bone metastasis showed an increase in RANKL and OPG. An unusually low TRACP-5b/RANKL ratio was observed in patients who had or later developed metastasis.
	Santini et al. (119)	Tumor tissue	74	66 patients (89%) with bone metastases were RANK-positive, and 40 patients (59.5%) showed >50% positive tumor cells. There was no significant difference between primary tumors and metastases.
	Ibrahim et al. (120)	Serum	49	RANKL was the most accurate marker, with an area under the curve of 0.74 (95% confidence interval: 0.54–0.93).
**CXCL12/CXCR4**	Zhou et al. (62)	Tumor tissue	150	The molecular model for predicting bone metastasis was logit (P) = −2.538 + 2.808 CXCR4 + 1.629 BSP + 0.846 OPN − 2.939 BMP4
	Li et al. (121)	Tumor tissue	65	Univariate analysis suggested that high expression of CXCR4 was significantly correlated with bone metastasis (p=0.004). In addition, it was marginally correlated with brain metastasis (p=0.068) and lymph node metastasis (p=0.085), as well as worse OS (p=0.004) and PFS (p=0.005).
	Liao et al. (122)	Tumor tissue	32	CXCR4 was highly expressed in the bone destruction area of metastatic NSCLC samples. Also, it was related to poor survival in NSCLC patients with bone metastasis, with an increase in VCAM1 and a decrease in ADAM17.
**C5A (CD88)/C5AR**	Ajona et al. (107)	Tumor tissue	95	Patients with high levels of C5AR1 had significantly shorter RFS and OS (p<0.001). They found significantly higher C5AR1 levels in primary NSCLC tumors from patients who developed bone metastases during disease progression compared with those from patients who developed metastases to other non-skeletal sites.

ADAM17, ADAM metallopeptidase domain 17; ALK, anaplastic lymphoma kinase; bmSUVmax, maximum standardized uptake value in bone metastasis; C5A, complement component 5a; C5AR, complement component 5a receptor; CXCL12, C-X-C motif chemokine ligand 12; CXCR4, C-X-C motif chemokine receptor 4; EGFR, epidermal growth factor receptor; F-FDG PET/CT, fluorodeoxyglucose positron emission tomography/computed tomography; KRAS, Kirsten rat sarcoma; mEGFR, EGFR mutation; NSCLC, non-small-cell lung cancer; OPG, osteoprotegerin; OS, overall survival; PFS, progression-free survival; RFS, recurrence-free survival; TRACP-5b, tartrate-resistant acid phosphatase isoform-5b; RANK, receptor activator of nuclear factor κB; RANKL, receptor activator of nuclear factor κB ligand; VCAM1, vascular cell adhesion molecule 1.

Most of the EGFR mutations are measured by tumor tissue immunohistochemistry through wounded puncture or surgery. Yao et al. (125) found a correlation between EGFR mutations and ^18^F-fluorodeoxyglucose positron emission tomography-computed tomography imaging, and attempted to use this non-invasive inspection as an independent predictor. It was also verified that circulating tumor DNA (ctDNA) in the plasma can be used to detect EGFR mutations in patients with NSCLC, providing information similar to that obtained from biopsies (126). Therefore, the predictive effect of EGFR mutations, ALK rearrangements, and KRAS mutations are with great clinical potential.

### C-Mesenchymal-Epithelial Transition Factor/Hepatocyte Growth Factor (c-MET/HGF) Signaling Axis

c-MET is a receptor tyrosine kinase, and HGF is its sole ligand. The dysregulation of c-MET and HGF in tumor progression and invasion interplay with other signaling pathways, such as the EGFR pathway (92, 118, 119). The prevalence of MET amplification and MET exon 14 skipping mutation (METex14) is approximately 5% and 2%, respectively.

In 2009, Navab et al. (120) performed a study involving the overexpression of c-MET and HGF, resulting in enhanced metastases to distant organs (e.g., bone, brain, and kidneys). Following a series of studies, c-MET was thought to be a biomarker for NSCLC, which could be measured by immunohistochemistry and qualified with the H-score ranging from 0 to 300. Tsakonas et al. (119) demonstrated that a c-MET H score ≥20 is a positive prognostic biomarker for OS in patients with early-stage NSCLC, and could also be used to predict the effect of platinum-based adjuvant chemotherapy. Notably, Grano et al. (127) conducted the first important study describing the expression and roles of c-MET and HGF in osteoblasts and osteoclasts. Moreover, Whang et al. (128) demonstrated that the c-MET and HGF signaling axis played an essential role in the metastatic bone microenvironments. Moreover, the expression of c-MET and HGF was correlated with the progression of bone metastasis. The expression of this axis was also viewed as a prognostic biomarker, representing the clinical response of patients with advanced lung cancer and prostate cancer to c-MET- and vascular endothelial growth factor receptor 2 (VEGFR2)-targeted therapy (92, 118, 129).

Currently, there is no approved therapy targeting c-MET, while several c-MET inhibitors (e.g., crizotinib, cabozantinib, tepotinib, telisotuzumab, etc.) are being investigated in clinical trials. **Table 4** presents the details of these clinical trials. According to a retrospective study, MET inhibitors are associated with prolonged OS of patients with metastatic METex14 NSCLC (130, 131). Secondary c-MET amplification is the most frequent cause of EGFR-TKIs tolerance. Following the administration of osimertinib as first-line therapy, MET amplification (encountered in 15% of patients) was the most common mechanism of intrinsic resistance (99). Moreover, >50% of the EGFR T790M-positive patients retained the c-MET amplification, and c-MET mutations are likely to be associated with EGFR C797S, CDK6, and BRAF amplifications (131, 132). Despite the absence of specific research regarding the predictive effects of c-MET and HGF, their predictive roles in the prognosis of patients with bone metastases are worthy of further investigation.

**Table 4:** Current studies on other markers.

Marker	Reference	Sample location	Sample size (N)	Finding
**CASR**	Liu et al. (174)	Tumor tissue	120	CASR expression in lung cancer tissues was significantly higher than that measured in adjacent and normal lung tissues. The expression of CASR in lung cancer tissues with BM was higher than that observed in non-metastatic lung cancer tissues.
**BSP**	Bellahcene et al. (33)	Primary lung tumor tissue	86	BSP was not specifically detected in normal lung tissue with the exception of cartilage associated with bronchi. Most adenocarcinoma (74%) and all squamous carcinoma of the lung samples examined exhibited detectable levels of BSP.
	Zhang et al. (70)	Primary lung tumor tissue	180	BSP protein expression in the primary resected NSCLC was strongly associated with BM and could be used to identify high-risk patients after primary tumor resection.
	He et al. (71)	Serum	146	The mean serum BSP levels in individuals with BM were significantly higher than those recorded in non-BM NSCLC and controls (p<0.001). The cut-off value was 33.56 ng/ml, and sensitivity and specificity values were 77.8% and 81.1%, respectively.
**BMP2**	Bieniasz et al. (168)	Tumor tissue		The expression levels of VEGF, BMP2, and BMP4 mRNA were significantly higher (7.1-fold, 25.6-fold, and 2.3-fold, respectively) in lung cancer samples than those in adjacent normal lung tissues.
	Choi et al. (169)	Serum	150	The NSCLC group demonstrated significantly higher levels of serum BMP2 than the control group. The median serum levels of BMP2 in the advanced stage group (stage IIIb or IV) were significantly elevated compared with those of the localized stage group (stages I, II, and IIIa).
	Fei et al. (170)	Serum	84	Serum BMP2 levels were significantly decreased in patients who achieved objective response after two cycles of chemotherapy.
	Huang et al. (171)	Tumor tissue	*in vivo* study	Activation of BMP2 signaling can enhance BM of Lewis lung carcinoma.
**CYFRA and CEA**	Numata et al. (182)	Serum/tumor tissue	131	Elevated serum CEA and CYFRA levels appear to provide useful clinical information on the presence of BM and liver metastasis, as well as multiple-organ metastases, although they were not a powerful indicator of prognosis.
**Tissue factor**	Xia et al. (186)	Serum	100	Patients with high tissue factor expression levels tended to have worse overall survival performance, and downregulation of tissue factor inhibited the invasion and metastasis of NSCLC cells *in vitro* and *in vivo*.
**Cell-free DNA (cfDNA)**	Pecuchet et al. (192)	Serum	124	The presence of circulating tumor DNA at baseline was an independent marker of poor prognosis.
	Ettinger et al. (83)	Serum	282	DNA median turnaround time was significantly shorter than that of tissue (9 vs. 15 days, respectively; p<0.0001)
	Ye et al. (193)	Tumor tissue	186	Patients with BM had higher concentrations of cfDNA and worse survival outcome.

BM, bone metastasis; BMP2, bone morphogenetic protein 2; BSP, bone sialoprotein; CASR, calcium sensing receptor; CEA, carcinoembryonic antigen; CYFRA, cytokeratin 19 fragment; NSCLC, non-small-cell lung cancer; VEGF, vascular endothelial growth factor.

### C-X-C Motif Chemokine Ligand 12/C-X-C Motif Chemokine Receptor 4 (CXCL12/CXCR4) Axis

CXCR4, a chemokine receptor, and its sole agonist CXCL12 [stromal cell-derived factor 1 (121)] play an important role in lung cancer. Their scavenger receptor, atypical chemokine receptor 3 (ACKR3; CXCR7), also participates in tumor progression.

Higher levels of CXCR4 are correlated with poor prognosis and a high risk of skeletal-related events in patients with NSCLC. Regarding patients with lung cancer, a univariate analysis conducted by Li et al. (133) suggested that high expression of CXCR4 was significantly associated with bone metastasis, as well as worse OS and PFS, and was marginally correlated with brain and lymph node metastasis. Zhou et al. (74) constructed a predictive model for bone metastasis, in which CXCR4 demonstrated an important role.

Several studies have shown that the CXCR4/CXCL12 interaction may foster local tumor growth and metastatic potential by maintaining an immune-quiescent microenvironment, which could be blocked by CXCR4 antagonism (134–138). It was also reported that the majority of NSCLC tumors expressed CXCL12 in the cytomembranous compartment, and strong staining for CXCL12 was associated with a higher incidence of disease recurrence (139). Other studies suggested that high CXCL12 expression in lymph nodes promoted the metastasis of CXCR4-expressing cells, benefiting from autocrine CXCL12/CXCR4 (121). Some meta-analyses have evaluated the whole effects of CXCL12/CXCR4 expression on NSCLC. The results supported that CXCR4 expression is correlated with lymph node metastasis, distant metastasis, tumor stage, and OS (140–142). Collectively, these studies proposed that CXCR4 is a predictive biomarker for bone metastasis and its prognosis.

The CXCL12/CXCR4 axis greatly contributes to certain tumor-mediated metastases and is thought to be a potential target of CXCR4 antagonists (143, 144). CXCR4 antagonists can be categorized into four major groups: nonpeptide CXCR4 antagonists (e.g., AMD3100); small peptide CXCR4 antagonists (e.g., BL-8040); antibodies to CXCR4 (e.g., LY 2624587 and ulocuplumab); and modified agonist CXCL12 antagonists (e.g., NOX-12A) (134, 145, 146). A series of clinical trials utilizing CXCR4 inhibitors were conducted in the field of hematological malignancies, while only a few clinical trials evaluated their safety and effectiveness in solid malignancies (147). Notably, small peptide LY2510924 exhibited limited clinical efficacy in the treatment of advanced SCLC (148). Therefore, it is essential to produce adequate evidence on the therapeutic role of CXCL12/CXCR4 in lung cancer and bone metastasis.

Moreover, the CXCL12/CXCR4 axis participates in other mechanisms of cancer progression. Recent findings indicated that CXCR4 expression on tumor cells plays a positive role in key downstream pathways, including PI3K/AKT/mechanistic target of rapamycin kinase (PI3K/AKT/MTOR) and extracellular signal-regulated kinase 1/2 (ERK1/2), which promote tumor proliferation and migration (149). Vascular cell adhesion molecule 1 (VCAM1) was also shown to be an activator of CXCR4, with ADAM metallopeptidase domain 17 (ADAM17) as a downstream mediator (122). Zuo et al. (150) demonstrated that overexpression of CXCR4 enhanced cell motility and invasion *via* EGFR and matrix metallopeptidase 9 (MMP9), owing to the positive correlation among them. Furthermore, CXCR4 regulates the migration of lung cells through activation of Rac family small GTPase 1 (RAC1) and matrix metalloproteinases (MMP2 and MMP14), and through the action of inhibitor of kappa kinase (IKK), ERK, NF-κB, and integrins (151, 152). Research has demonstrated the potential of CXCR4 antagonists to enhance the efficacy of immune checkpoint inhibitors in some types of solid tumor by inducing an immune suppressive tumor microenvironment (153–155). In summary, the predictive role of CXCL12/CXCR4 axis is reliable; however, the exact mechanism and therapeutic utilization of this axis warrant further verification.

### Complement Component 5a/Complement Component 5a Receptor (C5A/C5AR) Axis

The complement system represents a central component of innate immunity mediated by the proteolytic cleavage of C3 and C5. Cleaved products C3b and C5b participate in a variety of adaptive immune processes *via* membrane attack complex-dependent killing. Ajona et al. (107) demonstrated that blockage of the C5A/C5AR axis led to a substantial improvement in the efficacy of anti-programmed cell death 1 (anti-PD-1) immune responses in patients with lung cancer. Since then, C5AR-targeted therapy has been considered an advanced complementary therapy for clinical use. Furthermore, the C5A/C5AR axis is thought to be a reliable predictor of lung cancer bone metastasis. This is because patients who developed bone metastases had significantly higher C5AR1 levels in the primary tumor *versus* those with metastases to other non-skeletal sites during disease progression (108). To further investigate the mechanism of the C5A/C5AR axis, Vadrevu et al. (156) performed *in vitro* experiments and hypothesized that C5AR facilitates metastasis by suppressing effector CD8+ and CD4+ T-cells responses in the lungs. Recruitment of immature myeloid cells, generation of T regulatory cells, and secretion of factors, such as CXCL16, transforming growth factor beta (TGF-β), and interleukin 10 (IL10), may be involved in the mechanisms of this suppressor (108, 157).

### Notch Signaling Pathway

NOTCH3, a transmembrane receptor and a member of the Notch signaling pathway, plays an essential role in the development of lung cancer (158). It is overexpressed in approximately 40% of NSCLCs and has been associated with poorer disease-free survival and OS, higher TNM stage, poorer response to chemotherapy, and an increased rate of lymph node metastasis. Therefore, it is an ideal biomarker for the prediction of prognosis of NSCLC (105, 159–161). In NSCLC and SCLC, NOTCH3 signaling acts as a tumor-promoting and -suppressing pathway, respectively (162). The functional differences, which depend on the type of tumor, are worthy of exploration. Li et al. (163) found that upregulation of Wnt family member 3A (WNT3A) enhanced the expression of NOTCH3 and its downstream genes, hes family bHLH transcription factor 1 (HES1) and hes related family bHLH transcription factor with YRPW motif like (HEYL), and promoted metastasis of NSCLC. Liu et al. (164) demonstrated that NOTCH3 increased the invasion ability of NSCLC by upregulating zinc finger E-box binding homeobox 1 (ZEB1), which contributed to TGF-β-induced transformation and bone metastasis in NSCLC. High levels of NOTCH3 are also associated with chemoresistance and resistance to radiotherapy, and inhibition of Notch enhances the sensitivity to EGFR kinase inhibitor (159, 165). Notch signaling inhibitors, including MRK-003, represent new approaches for the targeted therapy for lung cancer. These inhibitors, to some extent, contribute to the diverse treatment options for bone metastasis (161). In summary, mRNA expression in tissue or immunohistochemistry staining of NOTCH3 from primary tumor tissue are likely to be stable predictive methods for prognosis and response to chemotherapy in patients with advanced NSCLC.

### CXCL16/CXCR6 Axis

CXCL16 is one of the most extensively studied transmembrane chemokines. It is lowly and highly expressed in lung tissues and specimens of bone metastasis, respectively (166). CXCR6 functions as the receptor of CXCL16, and the CXCL16/CXCR6 signaling system is involved in the progression of tumor growth. Ha et al. (166) demonstrated that high CXCL16/CXCR6 expression in prostate tumor tissue may be related to aggressive cancer behavior, as well as high CXCL16 expression in bone metastasis. Similarly, Na et al. (167) revealed that high mRNA expression levels and immunohistochemistry staining of CXCR6 and CXCL16 in Ewing sarcoma family tumors was associated with tumor progression and lung metastasis. Ajona et al. (108) found that CXCL16 participated in the blockage of the complement C5A/C5AR axis in lung cancer bone metastasis, suggesting that CXCL16 is also a potential biomarker for the prediction of lung cancer progression and bone metastasis.

## Other Potential Biomarkers

The bone formation and resorption markers have been used clinically for several years, based on their direct correlation with the progression of bone metastasis and clear mechanism. However, the efficacy and mechanism of some potential biomarkers remain unknown. Numerous studies have suggested their usefulness in the early prediction of bone metastasis and their clinical therapeutic or prognostic effect.

### Bone Morphogenic Protein 2 (BMP2)

BMP2 promotes the migration and invasion of NSCLC cells. Firstly, BMP2 expression is higher in tumor tissues than in adjacent normal lung tissues (168). Subsequently, Choi et al. (169) demonstrated significantly higher levels of serum BMP2 in patients with NSCLC *versus* the control group. In addition, the levels of BMP2 in the advanced stage group were significantly elevated compared with those measured in the localized stage group. Moreover, serum BMP2 has been strongly associated with the objective response to chemotherapy (170). Recently, Huang et al. (171) performed a series of *in vivo* experiments to certify that activation of BMP2 signaling can enhance bone metastasis of Lewis lung cancer through both osteolytic and osteoblastic mechanisms. Thus far, no retrospective research has directly analyzed the role of BMP2 in lung cancer bone metastasis. Nevertheless, it may be a useful noninvasive biomarker capable of prognostic utility.

### Calcium Sensing Receptor (CASR)

CASR is a widely expressed G protein-coupled receptor, which is critical for maintaining metabolic balance between bone and calcium content (172). Its expression is low or absent in normal lung tissue, whereas it is significantly higher in lung cancer tissue (173). Moreover, Lian et al. (174) demonstrated that the expression of CASR in 120 cancer tissues with bone metastasis was significantly higher than that recorded in non-metastatic lung cancer tissues. Another study found that the increase of CASR was the result of PTHrP and NF-κB upregulation, which was also positively correlated with bone metastasis in lung cancer (173, 175, 176). This evidence attracted attention to CASR as a new biomarker for the prediction of lung cancer bone metastasis.

### Sclerostin Domain Containing 1 (SOSTDC1)

SOSTDC1, secreted by particular helper T cells and reticular cells, plays a critical role in the development and progression of multiple types of cancer (177). Immunohistochemistry staining has indicated that SOSTDC1 is downregulated in NSCLC bone metastatic lesions compared with primary tumors (178). Moreover, Chen et al. (179) showed that overexpression of SOSTDC1 suppressed NSCLC migration, invasion, and osteoclast activity, whereas its knockdown led to the opposite effect. In addition, several downstream genes related to bone metastasis were detected using RNA-sequencing and quantitative RT-PCR (qRT-PCR) assays. Mechanistically, SOSTDC1 inhibits tumor progression by blocking the Wnt-β-catenin axis and facilitating T cell differentiation This finding suggests that SOSTDC1 is a potential prognostic biomarker for NSCLC bone metastasis (177).

### HECT and RLD Domain Containing E3 Ubiquitin Protein Ligase 5 (HERC5)

HERC5 is a gene fragment located in the chromosome 4q22 and associated with the early presence of disseminated tumor cells in the bone marrow. Accumulating evidence has shown that the HERC protein family is a key component of a wide range of cellular functions, including neurodevelopment, DNA damage repair, cell growth, and immune response (180). Wrage et al. (181) initially found the same loss of chromosome 4q12-q32 in brain metastases from lung cancer. Subsequently, they narrowed this loss to gene HERC5 using qRT-PCR. In addition, hypermethylation of the HERC5 promoter was associated with poor survival in patients with early-stage and metastatic lung cancer. Thus, HERC5 was thought to be a new metastasis suppressor gene, whose methylation and expression status may provide prognostic information for bone metastasis of NSCLC.

### Cytokeratin 19 Fragment/Carcinoembryonic Antigen (CYFRA/CEA)

CYFRA is one of the characteristic markers for the diagnosis of NSCLC. CEA is also widely used in clinical practice as a serum marker for NSCLC. Recently, Numata et al. concluded that elevated serum CEA and CYFRA levels were associated with the presence of bone and liver metastasis and also multiple-organ metastases; however, these high levels were not a powerful indicator of prognosis (38). Furthermore, serum CEA and CYFRA had predictive value with regard to response to therapy in NSCLC, and decreased levels of CYFRA are indicative of objective response (182). Hence, CEA and CYFRA were utilized in some clinical research studies as positive contrast to other markers (31, 35). The use of CEA and CYFRA presents opportunities for risk stratification of patients and may aid in the clinical management of the disease.

### Tissue Factor (TF)

TF, produced during the development of embryo and normal hemostasis, plays an essential role in regulating platelet activation, fibrin deposition, and the extrinsic coagulation cascade by binding to factor VIIa (183). It has been demonstrated that TF-induced fibrin deposition was positively associated with tumor progression by affecting complement activation and the recruitment of myeloid-derived suppressor cells (184). Xia et al. (185) reported that patients with high TF expression levels tended to have worse OS, and downregulation of TF inhibited the invasion and migration of NSCLC cells *in vitro* and *in vivo*. This evidence suggests that TF is an effective biomarker for predicting the prognosis of patients with NSCLC.

### Cell-Free DNA (cfDNA)

Nonencapsulated extracellular DNA fragments, termed cfDNA, have been found in body fluids. These fragments could also be narrowly defined as ctDNA in patients with solid malignancies (186, 187). cfDNA is thought to be generated during the process of cell apoptosis and necrosis, with a short half-life ranging 16 min–2.5 h. These features make it an ideal predictive biomarker for the early detection of lung cancer (188, 189). The use of cfDNA is attracting attention, as studies have shown that it can identify and differentiate the heterogeneous nature of different metastatic sites (190). Notably, higher concentrations of plasma cfDNA were detected in the peripheral blood of patients with bone metastasis. These higher levels are thought to be associated with an increased risk of distant migration and worse clinical outcome (191–193). Similarly, lung cancer patients without bone metastasis had significantly reduced urinary cfDNA and longer OS (194). Moreover, as the cfDNA turnaround time was significantly shorter than that of tissue-based genotyping, cfDNA was shown to be functional in the clinical management of the disease in the National Comprehensive Cancer Network (96).

In particular, cfDNA could be used as a non-invasive method to detect EGFR mutations in patients with NSCLC, with similar accuracy to that of tumor tissue biopsies. Dynamic changes in the cfDNA EGFR mutation status are associated with the clinical outcomes of treatment with EGFR-TKI (126, 195–199).

### Long Non-Coding RNA (lncRNA)

LncRNA are the products of DNA transcription with limited or no protein-coding capacities. However, lncRNA has a variety of functions in the regulation of biological processes, such as migration, proliferation, apoptosis, and invasion (200).

The expression levels of lncRNA in malignant tissues often differ significantly from those measured in normal tissues and correlate with tumor staging. Recently, studies utilizing high-throughput transcriptome analysis (RNA sequencing) indicated that lncRNA, including metastasis associated lung adenocarcinoma transcript 1 (MALAT1), PXN-AS1-L, and SUMO1 pseudogene 3 (SUMO1P3), etc., were significantly highly expressed in NSCLC tissues with bone migration. These findings suggested that lncRNA are of great predictive value in bone metastasis (201–203). However, the specific mechanisms involved in this relationship remain unknown and require further studies. Li et al. demonstrated that the plasma levels of lncRNA HOX transcript antisense RNA (HOTAIR) were higher in NSCLC samples *versus* normal specimens (204). Thus, lncRNAs can be utilized as biomarkers in NSCLC with or without bone metastasis.

### MicroRNA (miRNA)

MiRNAs are a class of small, single-stranded, no-coding RNAs, which were recently regarded a research hotspot. They act as gene regulator by binding to mRNAs and inhibiting their functions (205). Plenty of studies revealed that miRNAs play significant roles, including both oncogenes and tumor suppressors, during the progression and metastasis of lung cancer (206–208). Overexpression of the let-7 family, miR-486, miR-218, miR-34, and miR-200 were proved to inhibit the tumor cell proliferation, invasion and colony formation, though there is no specific article describing their relations with tumor cells bone migration (209–212). In the opposite, some miRNAs take oncogenic roles, containing miR-196b, miR-221/222, miR-21, miR17/92, and miR-224 (213–218). The genetic alterations or epigenetic changes of miRNAs are closely implicated in the metastasis of NSCLC, which identify more target therapies for lung cancer bone metastasis.

## Current and Next Generation Therapies

Currently, numerous promising drugs targeting biomarkers in patients with advanced/metastatic lung cancer carrying different mutations are under investigation. Clinical trials focusing on EGFR/ALK/KRAS, MET/HGF, and RANK/RANKL signaling have highlighted potential therapies for advanced NSCLC with or without distant metastasis. Ongoing clinical trials assessing the efficacy of EGFR inhibitors (afatinib, osimertinib, olmutinib, and poziotinib) and other newly developed drugs targeting ex20ins, T790, or other EGFR mutations are listed in **Table 5**. **Table 6** lists ongoing clinical trials assessing the efficacy of ALK inhibitors, including alectinib, brigatinib and lorlatinib which target advanced/metastatic ALK-positive NSCLC. **Table 7** shows two ongoing clinical trials of AMG510 and MRTX849 targeting lung cancer patients with the KRAS G12C mutation. **Table 8** summarizes information on ongoing clinical trials assessing the efficacy of MET inhibitors, such as cabozantinib, tepotinib, capmatinib, telisotuzumab, crizotinib, sacolitinib, and rilotumumab. **Table 9** presents the ongoing clinical trials involving patients with bone metastatic NSCLC treated with the RANK-targeting denosumab. To some extent, these clinical trials offer opportunities for late-stage patients and those who cannot afford curative tumor resections.

**Table 5:** Current studies on the efficacy of EGFR inhibitors in lung cancer.

Inhibitor	NCT number	Phase	Population	Treatment	Estimated enrollment	Primary outcome	Status
**Afatinib**	NCT03727724 (AFACET)	II	Advanced/metastatic NSCLC with EGFRex20ins	Afatinib 40 mg QD, cetuximab 500 mg/m2 iv Q2W	37	DCR	Recruiting
	NCT04206787(START)	NA	Advanced/metastatic NSCLC with EGFRm	Afatinib and 3rd generation EGFR TKI	825	TOT	Recruiting
	NCT03810872	II	Advanced/metastatic cancers harboring either an activating EGFR mutation or a HER2 mutation or a HER3 mutation	Afatinib 40 mg/day during Period 1 Afatinib 40 mg/day + Paclitaxel 80mg/kg/3w during Period 2	87	ORR and Incidence and intensity of adverse events	Recruiting
**Osimertinib**	NCT03414814 (KCSG LU17-19)	II	Pretreated advanced/metastatic NSCLC with EGFRex20ins	Osimertinib 80 mg QD	28	ORR	Active, not recruiting
	NCT02496663	I	Previously treated NSCLC with EGFR mutation including EGFRex20ins	Osimertinib 80 mg QD Necitumumab	100	Safety and tolerability	Recruiting
	NCT02151981	III	Locally advanced or metastatic NSCLC with T790M mutation	Osimertinib 80 mg QD	419	PFS	Recruiting
	NCT04035486(FLAURA2)	III	Advanced/metastatic NSCLC with EGFR mutation	Osimertinib 80mg QD	586	PFS	Recruiting
	NCT03460275	II	Advanced/metastatic NSCLC with EGFR mutation	Osimertinib Mesylate Tablets 80 mg QD	100	RECIST	Recruiting
	NCT03219970	NA	Locally advanced or metastatic NSCLC with T790M mutation	Osimertinib 80mg QD	156	OS	Active, not recruiting
	NCT03521154(LAURA)	III	Previously treated NSCLC with EGFR mutation	Osimertinib 80mg/40mg	200	PFS	Recruiting
	NCT02442349(AURA17)	II	Previously treated NSCLC with EGFR mutation	Osimertinib 80mg QD	171	ORR	Recruiting
**Avitinib**	NCT03856697	III	Advanced/metastatic NSCLC with EGFR mutation	Avitinib Maleate Capsules 300mg BID	406	PFS	Not yet recruiting
	NCT02274337	I/II	Previously treated advanced/metastatic NSCLC with EGFR mutation	Avitinib QD different dose stage	100	Safety, tolerability and ORR	Not yet recruiting
**Olmutinib (HM61713)**	NCT02485652	II	Previously treated NSCLC with EGFR T790M mutation	HM61713 800 mg QD	162	ORR	Active, not recruiting
**Poziotinib**	NCT04044170	II	Previously treated NSCLC with EGFRex20ins or HER2 ex20ins	Poziotinib orally	114	ORR	Active, not recruiting
**Mobocertinib (TAK-788)**	NCT03807778	I/II	Previously treated NSCLC with EGFRex20ins	TAK-788 40mg QD phase 1, TAK-788 160 mg QD phase 2	63	ORR	Recruiting
**Pyrotinib**	NCT04063462	II	Previously treated NSCLC with EGFRex20ins or HER2 ex20ins	Pyrotinib 400 mg QD	60	ORR	Not yet recruiting
	NCT03574402 (TRUMP)	II	Arm9: NSCLC with EGFRex20ins	Pyrotinib either 60 mg QD or 40 mg BID	400	ORR	Recruiting
**Tarloxotinib (TH-4000)**	NCT03805841 (RAIN)	II	NSCLC with EGFR ex20ins or HER2 activating mutation	Tarloxotinib bromide	60	ORR	Recruiting
**DZD9008**	NCT03974022	I/II	Advanced/metastatic NSCLC with EGFR/HER2 mutations	DZD9008	160	ORR	Recruiting
**Lazertinib (YH25448)**	NCT04248829 (Lazertinib)	III	Locally advanced or metastatic NSCLC with EGFR mutation	Lazertinib 240 mg/160 mg	380	PFS	Recruiting
**JNJ-61186372**	NCT04077463	I/II	Previously treated NSCLC with EGFR mutation	Lazertinib and JNJ-61186372	120	DLT and ORR	Recruiting
	NCT02609776	I	Previously treated NSCLC with EGFR mutation	JNJ-61186372 140 mg	460	DLT and ORR	Recruiting

NSCLC, non-small cell lung cancer; EGFR, epidermal growth factor receptor; Ex, exon; Ins, insertion; HER2, human epidermal growth factor receptor2; Mut, mutation; QD, once daily; BID, twice daily; DCR, disease control rate; ORR, objective response rate; OS, overall survival; PFS, progression free survival; RECIST, response evaluation criteria in solid tumors; TOT, time on treatment; DLT, dose limiting toxicity. NA, missing value.

**Table 6:** Ongoing clinical trials assessing the efficacy of ALK inhibitors in lung cancer.

Inhibitor	NCT number	Phase	Population	Treatment	Estimated enrollment	Primary outcome	Status
***Alectinib***	***NCT02075840***	III	Untreated NSCLC with ALK mutation	Alectinib 600mg BID	303	PFS	Active, not recruiting
	***NCT02838420***	III	Advanced/metastatic ALK-positive NSCLC	Alectinib 600mg BID	187	PFS	Active, not recruiting
	***NCT03456076***	III	Advanced/metastatic ALK-positive NSCLC	Alectinib 600mg BID	255	DSF	Recruiting
***Brigatinib***	***NCT03535740 (ALTA-2)***	II	Advanced/metastatic ALK-positive NSCLC	Brigatinib 90mg QD	104	ORR	Active, not recruiting
	***NCT03596866 (ALTA-3)***	III	Advanced/metastatic ALK-positive NSCLC	Brigatinib 90mg QD	246	PFS	Recruiting
***Lorlatinib***	***NCT03052608***	III	Advanced/metastatic ALK-positive NSCLC	Lorlatinib 100 mg QD	280	PFS	Recruiting
	***NCT03909971***	II	Advanced/metastatic ALK-positive NSCLC	Lorlatinib 100 mg QD	100	OR	Recruiting

NSCLC, non-small cell lung cancer; ALK, anaplastic lymphoma kinase; QD, once daily; BID, twice daily; ORR, objective response rate; OS, overall survival; PFS, progression free survival; DSF, disease-free survival.

**Table 7:** Ongoing clinical trials assessing the efficacy of KRAS inhibitors in lung cancer.

Inhibitor	NCT number	Phase	Population	Treatment	Estimated enrollment	Primary outcome	Status
**AMG 510**	NCT04303780	III	Advanced/metastatic NSCLC with KRAS G12C mutation	AMG 510	650	PFS	Recruiting
**MRTX849**	NCT04330664	I/II	Solid tumor malignancy with KRAS G12C mutation	MRTX849	148	Safety and pharmacokinetics	Recruiting

NSCLC, non-small cell lung cancer; KRAS, kirsten rat sarcoma mutations; PFS, progression free survival.

**Table 8:** Ongoing clinical trials assessing the efficacy of MET inhibitors in lung cancer.

Inhibitor	NCT number	Phase	Population	Treatment	Estimated enrollment	Primary outcome	Status
***Cabozantinib (XL184)***	*NCT01639508*	II	Group B includes MET amplification	Cabozantinib 60mg QD	68	ORR	Recruiting
	*NCT03911193*	II	Advanced lung cancer with MET mutations	Cabozantinib 60mg QD	25	ORR	Recruiting
***Tepotinib***	*NCT02864992(VISION)*	II	Advanced NSCLC with METex14 or MET amplification	Tepotinib 500mg QD	330	ORR	Recruiting
	*NCT03940703(INSIGHT 2)*	II	Locally advanced or metastatic NSCLC with MET amplification		120	DLTs and ORR	Recruiting
***Capmatinib (INC280)***	*NCT03693339*	II	Advanced NSCLC with METex14	Capmatinib 400 mg TID	27	ORR	Recruiting
	*NCT02414139*	II	Previously treated c-MET+ NSCLC	INC280 (capmatinib)	368	ORR	Recruiting
	*NCT02750215*	II	Previously treated NSCLC with MET mutation	INC280 (capmatinib)	20	ORR	Active, not recruiting
***Telisotuzumab (ABBV-399)***	*NCT03539536*	II	Previously treated c-MET+ NSCLC	Telisotuzumab	310	ORR	Recruiting
***Crizotinib***	*NCT04084717*	II	Advanced NSCLC with MET mutation	Crizotinib 250mg BID	50	ORR and PFS	Recruiting
***Savolitinib***	*NCT03778229*	II	Advanced NSCLC with EGFRm+ MET+	Savolitinib 300 mg or 600 mg QD	192	ORR	Recruiting
***Rilotumumab (AMG-102)***	*NCT02154490*	II/III	S1400E Arm I: SCCA with HGF/c-MET positive	Rilotumumab IV and erlotinib daily	10000	PFS, ORR and OS	Active, not recruiting

NSCLC, non-small cell lung cancer; MET, mesenchymal-epithelial transition factors; Ex, exon; Mut, mutation; QD, once daily; BID, twice daily; TID, third daily; ORR, objective response rate; OS, overall survival; PFS, progression free survival; DLT, dose limiting toxicity.

**Table 9:** Ongoing clinical trials assessing the efficacy of RANK inhibitors in lung cancer.

Inhibitor	NCT number	Phase	Population	Treatment	Estimated enrollment	Primary outcome	Status
***Denosumab***	*NCT03669523*	II	NSCLC with bone metastasis	Denosumab+nivolumab	86	ORR	Recruiting
	*NCT03958565*	NA	Pathologically confirmed NSCLC with bone metastasis but no driver oncogene	Denosumab 120 mg	100	Percentage reduction of urine NTx and serum CTx	Recruiting

NSCLC, non-small cell lung cancer; RANK, receptor activator of nuclear factor kB; ORR, objective response rate.NA, missing value.

There is no approved therapy targeting c-MET, while several c-MET inhibitors (e.g., crizotinib, cabozantinib, tepotinib, telisotuzumab, etc.) are being investigated in clinical trials. **Table 10** presents the details of these clinical trials. According to a retrospective study, MET inhibitors are associated with prolonged OS of patients with metastatic METex14 NSCLC (130, 131). Secondary c-MET amplification is the most frequent cause of EGFR-TKIs tolerance. Following the administration of osimertinib as first-line therapy, MET amplification (encountered in 15% of patients) was the most common mechanism of intrinsic resistance (99). Moreover, >50% of the EGFR T790M-positive patients retained the c-MET amplification, and c-MET mutations are likely to be associated with EGFR C797S, CDK6, and BRAF amplifications (131, 132). These phenomenon provide opportunities for the combined treatment of lung cancer patients.

**Table 10:** Current studies on other markers.

Marker	Reference	Sample location	Sample size (N)	Finding
**CASR**	Liu et al. (174)	Tumor tissue	120	CASR expression in lung cancer tissues was significantly higher than that measured in adjacent and normal lung tissues. The expression of CASR in lung cancer tissues with BM was higher than that observed in non-metastatic lung cancer tissues.
**BSP**	Bellahcene et al. (33)	Primary lung tumor tissue	86	BSP was not specifically detected in normal lung tissue with the exception of cartilage associated with bronchi. Most adenocarcinoma (74%) and all squamous carcinoma of the lung samples examined exhibited detectable levels of BSP.
	Zhang et al. (70)	Primary lung tumor tissue	180	BSP protein expression in the primary resected NSCLC was strongly associated with BM and could be used to identify high-risk patients after primary tumor resection.
	He et al. (71)	Serum	146	The mean serum BSP levels in individuals with BM were significantly higher than those recorded in non-BM NSCLC and controls (p<0.001). The cut-off value was 33.56 ng/ml, and sensitivity and specificity values were 77.8% and 81.1%, respectively.
**BMP2**	Bieniasz et al. (168)	Tumor tissue		The expression levels of VEGF, BMP2, and BMP4 mRNA were significantly higher (7.1-fold, 25.6-fold, and 2.3-fold, respectively) in lung cancer samples than those in adjacent normal lung tissues.
	Choi et al. (169)	Serum	150	The NSCLC group demonstrated significantly higher levels of serum BMP2 than the control group. The median serum levels of BMP2 in the advanced stage group (stage IIIb or IV) were significantly elevated compared with those of the localized stage group (stages I, II, and IIIa).
	Fei et al. (170)	Serum	84	Serum BMP2 levels were significantly decreased in patients who achieved objective response after two cycles of chemotherapy.
	Huang et al. (171)	Tumor tissue	*in vivo* study	Activation of BMP2 signaling can enhance BM of Lewis lung carcinoma.
**CYFRA and CEA**	Numata et al. (182)	Serum/tumor tissue	131	Elevated serum CEA and CYFRA levels appear to provide useful clinical information on the presence of BM and liver metastasis, as well as multiple-organ metastases, although they were not a powerful indicator of prognosis.
**Tissue factor**	Xia et al. (186)	Serum	100	Patients with high tissue factor expression levels tended to have worse overall survival performance, and downregulation of tissue factor inhibited the invasion and metastasis of NSCLC cells *in vitro* and *in vivo*.
**Cell-free DNA (cfDNA)**	Pecuchet et al. (192)	Serum	124	The presence of circulating tumor DNA at baseline was an independent marker of poor prognosis.
	Ettinger et al. (83)	Serum	282	DNA median turnaround time was significantly shorter than that of tissue (9 vs. 15 days, respectively; p<0.0001)
	Ye et al. (193)	Tumor tissue	186	Patients with BM had higher concentrations of cfDNA and worse survival outcome.

BM, bone metastasis; BMP2, bone morphogenetic protein 2; BSP, bone sialoprotein; CASR, calcium sensing receptor; CEA, carcinoembryonic antigen; CYFRA, cytokeratin 19 fragment; NSCLC, non-small-cell lung cancer; VEGF, vascular endothelial growth factor.

For the future, therapies utilizing lncRNA is under development, which works by affecting transcriptome directly. Studies suggested that lncRNA was associated with resistance to both chemotherapy and targeted therapies (219). For example, lncRNA LINC00460 promotes EGFR expression by downregulating miR-769-5p, which results in the resistance of NSCLC cells to gefitinib (220). LncRNA MALAT1 upregulates multiple drug resistance-related protein (MDR1) and multidrug resistance protein (MDR1) by inducing STAT3 phosphorylation, which eventually enhance the resistance of lung cancer cells to treatment with cisplatin (221, 222). Though there is no specific clinical trial targeting lung cancer, it’s still a potential therapy for the comprehensive treatment of lung cancer.

## Conclusion

Patients with lung cancer bone metastasis rarely receive effective targeted therapy at an early stage due to delayed diagnosis. Therefore, the discovery of more accurate diagnostic methods is warranted. The bone formation and resorption markers have already been applied to clinical practice, and greatly aid in improving clinical management. For example, liquid biopsy of circulating tumor cancer, circulating cell-free nucleic acids, and extracellular vesicles are a promising source of prognostic and therapeutic biomarkers for metastatic lung cancer (223–225). However, thus far, most of the signaling axes and other markers remain under investigation without a recognized standard due to inconsistent results and methodological differences. To combine the advantages of these markers, some research studies utilized meta-analysis to identify clinical criteria and construct a clinical model. However, the limited number of samples and marker types restrict the reliability of this model (32, 52, 74). Consequently, it is necessary to integrate clinical resources and utilize larger sample sizes, as well as unified standards for the selection of patients and evaluation of bone metastasis and clinical outcomes. This approach would allow researchers to determine the ideal, inexpensive biomarkers for the evaluation of skeletal metastasis and skeletal-related events. Moreover, combination therapies targeting these biomarkers are currently under investigation in clinical trials. Based on the available evidence regarding these biomarkers, the prognosis of treatment using targeted therapies is promising. Further studies concerning the mechanisms of these biomarkers will contribute to the development of effective therapies against bone metastasis.

## Author Contributions

XC, EY and ZeW wrote the manuscript. ZhW, BL, and YZ provided expertise and advice. HZ, YWE, SW and WZ critically read the manuscript. ZY supervised the project. All authors contributed to the article and approved the submitted version.

## Funding

This work was supported by National Natural Science Foundation of China (81872173, 82072959); Natural Science Foundation of Zhejiang province, China (LD21H160002); Medical and Health Science and Technology Plan of Department of Health of Zhejiang Province (WKJ-ZJ-1821).

## Conflict of Interest

The authors declare that the research was conducted in the absence of any commercial or financial relationships that could be construed as a potential conflict of interest.

## Publisher’s Note

All claims expressed in this article are solely those of the authors and do not necessarily represent those of their affiliated organizations, or those of the publisher, the editors and the reviewers. Any product that may be evaluated in this article, or claim that may be made by its manufacturer, is not guaranteed or endorsed by the publisher.
